# Will the Volume-Based Procurement Policy Promote Pharmaceutical Firms’ R&D Investment in China? An Event Study Approach

**DOI:** 10.3390/ijerph182212037

**Published:** 2021-11-16

**Authors:** Yuanyuan Hu, Shouming Chen, Fangjun Qiu, Peien Chen, Shaoxiong Chen

**Affiliations:** 1 School of Economics and Management, Tongji University, Shanghai 200092, China; huyuanyuan@tongji.edu.cn (Y.H.); schen@tongji.edu.cn (S.C.); 2 Eli Broad College of Business, Michigan State University, East Lansing, MI 48824, USA; 3 Shanghai Biopharmaceutics Industry Association, Shanghai 201203, China; sxchen@sbia.org.cn

**Keywords:** innovation, pharmaceutical firms, firm value, R&D intensity, “4 + 7” volume-based procurement policy, event study, DiD

## Abstract

Innovation is the key to the development of the pharmaceutical industry. The pilot program of China’s “4 + 7” volume-based procurement policy (“4 + 7” procurement policy) brings the drug price back to a reasonable level through trading procurement quantities for lower drug prices. The policy manages to reduce the burden of the health care system, improve efficiency, and push the pharmaceutical industry to transform and update from the era of high gross profit of generic drugs to innovative drugs. So far, few studies have investigated the influence of the volume-based procurement policy on the innovation of pharmaceutical firms. By combining the event study and Difference-in-Difference (DiD) methodology, this study finds that the abnormal return (AR) of firms with high R&D intensity is lower than that of firms with low R&D intensity during the event window period. Moreover, further analysis identifies the moderating effect of firm size and firm type. Specifically, the results show that the negative influence of high R&D intensity on abnormal return (AR) during the announcement of the “4 + 7” procurement policy is stronger in large firms and innovative pharmaceutical firms. Finally, we discuss the policy implications of our study.

## 1. Introduction

Since the outbreak of the COVID-19 pandemic, people have been looking forward to the invention of vaccines and specific treatments, which relies on innovation. Encouraging innovation and R&D investment of pharmaceutical firms has become a consensus among governments around the world ever since. In the pandemic, the thirst for a “savior” and the foreseeable profits have made the pharmaceutical industry widely concerned by all walks of life [1,2,3].

In the innovation-intensive medical field, the public procurement department is always the primary user of the innovation results [1,2] while innovations are often by-products during achieving procurement policy objectives [4]. Although there may be serious tensions between procurement policy objectives and innovation policy objectives, pursuing the former could now and then enhance the latter. [5]. The recent “innovation policy directionality” also once again provides evidence that procurement policies could promote innovation [5]. For example, in developed countries, there are increasing numbers of public procurement of innovation (PPI) policies [2,6], aimed at encouraging firms to research and development (R&D), induce or accelerate diffusion, improve public services, or achieve key public policy objectives [4,5,7].

In practice, in addition to a small number of PPI policies, the centralized procurement policy is the internationally common practice in public procurement in the field of medicine [3,8,9,10,11]. The centralized procurement policy can reduce costs, improve efficiency, control drug prices, and ensure drug supply. Furthermore, there are various centralized procurement modes in different countries, such as third-party group procurement mode, medical institution alliance procurement mode, government direct procurement mode, etc. [10,11,12,13,14,15]. Among them, China mainly adopts the government direct procurement mode in medical procurement [8,16,17,18]. Since the end of 2018, China has piloted the volume-based procurement policy in 11 key cities, known as the “4 + 7” procurement policy [19,20]. After the announcement of this policy, drug prices have witnessed significant reduction [18,21]. Afterwards, the policy was continuously promoted. From the first expansion of volume procurement in September 2019 to the official announcement of the bidding results of the fourth batch of national drug volume-based procurement in February 2021 [22], the “4 + 7” procurement policy has been promoted and implemented to the whole of China. Since then, some pharmaceutical stocks have plummeted frequently, causing big shocks in the whole industry, indicating that the pharmaceutical industry has started the wave washing sand model and many firms will be washed out of the market [8,17]. In this context, it is of great practical significance to study the impact of the centralized procurement policy on the pharmaceutical industry [8,9,17,23,24].

The impact of public procurement policy on innovation has attracted more and more attention in academia [1,4,7,25]. China’s new volume-based procurement policy is not only to reduce the drug cost but also to try to slash margins of the drugs by centralized procurement of the generic drugs, and thus to promote the development of innovative drugs. However, the studies after the release of the centralized procurement policy mainly focus on the impact of the policy on drug prices and patient medication [14,16,18,21,26,27,28,29]. Furthermore, few theoretical and empirical studies have systematically investigated the relationship between the centralized procurement policy and the innovation of the pharmaceutical industry [7].

In conclusion, our study is to investigate the following question: Will the implementation of the “4 + 7” procurement policy promotes the transformation and upgrading of the Chinese pharmaceutical manufacturing industry from the era of generic drugs to innovative drugs? In other words, will the “4 + 7” procurement policy promote or inhibit the R&D investment of pharmaceutical firms?

To answer the above questions, based on analyses of a sample of A-share pharmaceutical listed companies in Shanghai and Shenzhen stock markets, this study employs event study and Difference-in-Difference (DiD) methodology to test the influence of R&D investment intensity of pharmaceutical firms on negative market reaction in the context of China’s pilot “4 + 7” volume-based procurement policy in December 2018. Our study contributes in three ways. First, this paper expands the procurement policy literature by empirically testing the influence of the “4 + 7” procurement policy on the R&D investment of pharmaceutical firms for the first time. Second, we employ event study and PSM-DID methodology to alleviate the endogenous problems of general regression analysis to a certain extent and make the research conclusion more credible. Finally, this study also contributes to the innovation studies by putting forward the mitigation solutions for the potential negative impact of “4 + 7” procurement policy casts on R&D investment in the pharmaceutical industry.

## 2. Theory and Hypothesis

### 2.1. “4 + 7” Volume-Based Procurement Policy

As mentioned above, centralized procurement is the common practice worldwide [3,8,9,10,30]. There are various procurement modes in different countries, such as third-party group procurement, medical institution alliance procurement, and government direct procurement [10,11,12,13,14,15]. Moreover, China mainly employs government direct procurement [8,16,17,18]. The industry generally believes that China’s new “4 + 7” procurement policy is designed to bring down drug prices and improve drug quality, to upgrade the pharmaceutical industry and deepen the reform of public hospitals, and to ease the burden of the healthcare system [9,11,16].

The “4 + 7” procurement policy refers to the volume-based procurement policy piloted in the four municipalities (Beijing, Shanghai, Tianjin, and Chongqing) and seven sub-provincial cities (Guangzhou, Shenzhen, Shenyang, Dalian, Xi’an, Chengdu, and Xiamen). The whole procurement process goes as follows. First, public hospitals in pilot cities draft the purchase list and estimate the purchase volume according to 60% to 70% of the total drug consumption of the previous year. Then, taking the procurement volume as a chip, the healthcare department determines the purchase variety and volume, holds a meeting, and negotiates with the pharmaceutical firms. Last, the firm which gives the lowest price wins the bid.

The “4 + 7” procurement policy manages to exchange volume for a lower price through the spillover effect of competition, economies of scale, and transaction cost saving (search costs, supervision costs, and negotiation costs). Some studies show that since the implementation of the policy, remarkable results have been achieved in various aspects, such as significant drug price reductions, fairer competitions among firms, the upgrading of the pharmaceutical industry, the better linkage of the three medical institutions and reforms in related fields [16,17,21,29].

### 2.2. R&D Investment and Market Reaction during the Introduction of the “4 + 7” Procurement Policy

Some scholars propose that whether the government has planned a clear innovation-oriented procurement policy or not, their decisions on price, volume, and standard will have a positive or negative impact on innovation of corresponding industries participating in government procurement [4,31,32]. The question of whether the “4 + 7” volume-based procurement policy promotes or inhibits the R&D investment of pharmaceutical firms can be transformed into the following question: does the high R&D investment alleviate or aggravate the negative market reaction during the announcement of “4 + 7” volume-based procurement policy?

Most views suggest that China’s “4 + 7” volume-based procurement policy targets knocking down the high profits of generic drugs. Firstly, from the perspective of industrial economics, this policy may negatively influence small and medium-sized generic drug firms, especially those with weak R&D capabilities by increasing entry barriers. In other words, this policy provides opportunities for firms with scale advantages and strong R&D capabilities.

Secondly, according to the theory of contestable markets [33], the “4 + 7” volume-based procurement policy may force the firms profiting from increasing the turnover of generic drugs to shift from the sales-centered mode to the R&D-centered mode by increasing the external contestability of the pharmaceutical market. Specifically, the “4 + 7” volume-based procurement policy adopts the standard of winning the bid at the lowest price under the condition of consistency. On the one hand, the policy forms price competition and quickly compresses the overall profit margin of the generic pharmaceutical industry. On the other hand, with the preference for low prices heating up the competition in the generic drug industry, it brings opportunities for drugs with low domestic market share—the drugs listed in procurement can directly occupy more than 60% of the market of pilot cities, indicating the transition from the era of high gross profit of generic drugs to the innovative drugs. In other words, firms with low R&D investment are more impacted by the “4 + 7” volume-based procurement policy.

Finally, the volume procurement policy adopts price negotiation for patented drugs and exclusively produced drugs, which also means that technological innovation and technological progress will become the core factor of firm competitiveness in the future. In other words, firms with high R&D investment are theoretically less affected by the “4 + 7” volume-based procurement policy. In short, the volume procurement policy once again emphasizes the long-term value of R&D investment to the market.

In conclusion, investors may consider companies with high R&D investment intensity responding better and faster to the “4 + 7” procurement policy when the policy was announced. These conclusions lead us to hypothesize the following:

**Hypothesis** **a.** *Compared with firms with low R&D investment intensity, firms with high R&D investment intensity suffer less firm value loss caused by the “4 + 7” volume-based procurement policy.*

However, from another perspective, no matter whether the firm wins the bid, whether the firm locates in the pilot area, and whether the firm has drugs that pass the consistency evaluation, the price reduction effect will eventually form the domino effect in the whole industry after the implementation of the “4 + 7” volume procurement policy due to the large market share of the bid winning drugs. In this way, cost control will become the key to the survival and development of pharmaceutical firms.

As R&D investment requires heavy investments, takes long periods, and faces high risks, the “4 + 7” volume-based procurement policy may have an adverse impact on the firm value by highlighting the high cost, information asymmetry and agency problems. These problems can have a negative influence on the firm value. First, in the context of the “4 + 7” volume-based procurement policy, high R&D investment may become a kind of “liability”, while firms with low R&D investment could adjust costs flexibly and respond strategically. The “4 + 7” volume-based procurement policy reduces drug prices by linking the purchase volume with the price. When the price of drugs drops sharply, the firms with high R&D investment may have to give up the chance of winning the bid because they have difficulties covering the cost in a short time, and thus they lose the market. In addition, even if the firms may lower the price to participate in bidding to occupy the market share, their profit will be inevitably affected and the risk of insolvency will increase because of the sharp increase in cost, especially when the firms invest heavily in R&D.

Secondly, the problem of information asymmetry between managers and investors caused by R&D investment will be more prominent in the context of volume-based procurement. Previous studies have shown that in firms with high R&D investment intensity, the information asymmetry between investors and managers is more serious because of the strong professionalism in R&D investment [34,35]. Especially under the context of the industry earthquake caused by “4 + 7” volume-based procurement, the neglected information asymmetry contradiction may be amplified.

Last but not least, in terms of the principal-agent problem, the agency theory insists that managers have the motivation to conduct investment activities for their own interests. The high-risk characteristic of R&D investment makes it vulnerable to problems of low investment efficiency like over-investment and insufficient investment. Studies have found that some companies made R&D investments intentionally in order to meet the standards of government preferential policies, resulting in excessive R & D investment and investment inefficiency [36,37]. The negative impact of the above motivation is more like to be exposed in the case of an industry earthquake caused by the “4 + 7” volume-based procurement policy. When the accumulation of negative impact exceeds a certain critical value, it may eventually affect the firm value. In general, under such circumstances, although the “4 + 7” procurement policy eliminates institutional costs and marketing costs for firms, it induces investors to pay more attention to the cost problems faced by firms and the previously neglected corporate governance problems.

In conclusion, we believe that high R&D intensity firms are vulnerable to potential negative influences in the context of the “4 + 7” procurement policy. The conclusion leads us to hypothesize the following:

**Hypothesis** **b.** *Compared with firms with low R&D investment intensity, firms with high R&D investment intensity suffer more loss caused by the “4 + 7” volume-based procurement policy.*

## 3. Research Design

This study aims to investigate how high R&D intensity influences firm value before and after the “4 + 7” procurement policy. We employ the event study and the DiD methodology to test our hypotheses. According to the efficient market hypothesis of Eugene F. Fama, all valuable information can be precisely and timely reflected through the stock price [38]. In this way, we can observe stock price fluctuations during the event window to analyze the influence of the event, just as many studies already have done in investigating how the outbreak of events influence firm value [39,40,41]. The DiD methodology avoids endogeneity raised by reverse causality and omitted variables. By setting high or low R&D intensity and before-and-after-event as dummy variables, the DiD methodology analyzes the influence of high R&D intensity on firm value more specifically.

### 3.1. Sample Selection and Data

We selected A-share pharmaceutical listed companies from Shanghai Stock Exchange and Shenzhen Stock Exchange of the year 2018 as samples. We performed the selection by excluding ST and *ST companies (which are about to delist) and companies with insufficient trading data during the event window and the estimation window.

We obtained the stock market information, CSI 300 Index and other firm-level data from the China Stock Market Accounting Research (CSMAR) database and WIND database which contained the most comprehensive information about domestic firms in China. Both databases have been used by a number of researchers and exhibit robust construct validity around its underlying measures [42]. Then we obtained the bid information of pharmaceutical enterprises in procurement from the public documents released on the Sunshine Medical Procurement All-In-One website. Finally, after we combined the datasets and removed observations with missing key variables, our final full sample consists of 3162 samples observed in 21 days for 205 companies.

### 3.2. Variables

#### 3.2.1. Dependent Variable

- Firm value

Based on previous literature [41], we conducted the event study and measured firm value by calculating the cumulative abnormal return (CAR) and abnormal return (AR) surrounding the event of the “4 + 7” procurement policy. The measurement includes the following steps.

- Event day

We searched for related news about the “4 + 7” procurement policy. The first mention of it was on 14 November 2018, in the document entitled National Centralized Drug Procurement Pilot Plan, approved at the fifth meeting of the Central Committee for Comprehensive Deepening Reform [43]. The document clarifies the overall policy mechanism design [43]. Then on 15 November, the Joint Procurement Office formed by the 11 pilot cities released 4 + 7 City Drug Centralized Procurement Documents, clarifying the working body and procurement catalogs, but did not mention anything concerning firm participation [19]. These two dates released no specific information for firms so the market was still unable to predict or react to the new policy at that time [44]. Varied predictions appeared. Some thought that the “4 + 7” procurement policy could save the marketing cost for pharmaceutical firms. Some thought that the bid-winning one would squeeze out the market share of the bid-off ones and bankrupt them. Moreover, the price reduction was unknown despite “4 + 7” procurement policy emphasized “volume for price”. All in all, these two dates when official documents were released cannot be regarded as the event day.

The third time point related to the “4 + 7” procurement policy is the day when the pre-selected and selected lists of centralized drug procurement were published. On 6 December 2018, the pre-selected list was published and the newly released floor prices knocked down the stock prices of multinational pharmaceutical companies immediately. As pre-selection was not the final result, it was still not clear if domestic pharmaceutical companies would face the same price reduction. On 7 December the website Sunshine Medical Procurement All-In-One officially published the first selected list of the “4 + 7” centralized procurement [45]. The outcome listed the winning companies and prices of procured medicines in detail. Upon publishing, the market knew how pharmaceutical companies would be influenced. The price reduction as released on 7 December was beyond prediction and on the same day, the domestic pharmaceutical companies witnessed a drop in stock prices. Taking both the influence made by the outcome on 6 and 7 December into consideration, we took 7 December 2018 as the event day (t = 0).

- Event window

Considering confounding effects and possible information leaks, the event window is usually limited to a relatively short period. Based on previous literature [46], we set the 10 days before and after 7 December 2018 (21 days in total) as the event window. Estimation window is 250 days before the event window. Taking 7 December as the event day (t = 0), the event window is (−10,10) and the estimation window is (−260,−11).

- Estimation model

Following previous literature [47], we employed the market model and regressed firms’ returns on the stock market against the return of market portfolio during the estimation model to estimate the CAR.

The market model is set as Equation (1) below:

(1) $$R_{it}=\alpha_{i}+\beta_{i}R_{mt}+\varepsilon_{it}$$

In model (1), $R_{it}$ is the stock return of the *i*th firm on trading day *t*; $R_{mt}$ is the market return on trading day *t*; $\varepsilon_{it}$ is the abnormal return of the *i*th firm on trading day *t*. $\alpha_{i}$ captures the risk-free return rate of the *i*th firm and $\beta_{i}$ captures the *i*th firm’s movement of return relative to the market risk. We estimate the two return factors ${\hat{\alpha }}_{i}$ and ${\hat{\beta }}_{i}$ over the estimation window (−11,250) using least square regression, and based on that we estimate the expected return of the *i*th firm during the event window (−10,10) as below:

(2) $$E\left[R_{it\left(event\right)}\right]={\hat{\alpha }}_{i}+{\hat{\beta }}_{i}R_{mt\left(event\right)}$$

The AR is the difference between the real return and the estimated return during the event window:

(3) $$AR_{it}=R_{it}-\left({\hat{\alpha }}_{i}+{\hat{\beta }}_{i}R_{mt\left(event\right)}\right)$$

- Significance test

The significance test to the CARs of samples prepares for further modeling and analysis. The t-test results are shown in Table 1. Despite different event windows, the coefficients of CAR all appear significant, indicating that the “4 + 7” procurement policy cast significant influence on the firm value of pharmaceutical firms. Furthermore, all the coefficients of CAR are significantly negative (−5.78%, *p* < 0.001), supporting the hypothesis that the “4 + 7” procurement policy dealt a blow to the pharmaceutical industry.

#### 3.2.2. Independent Variables

China began to implement the “4 + 7” procurement policy in 2018 and the bidding result was published on 7 December 2018. To discuss the influence of “4 + 7” procurement policy on firms with different R&D investment intensities, we divided the samples into the control group and the treatment group according to R&D intensity. Following previous literature [48], we defined firms with R&D intensity above 75% quantile as high R&D investment firms and used a dummy variable (Treat) to proxy that. High R&D investment firms belong to the treatment group, while low R&D investment firms belong to the control group. For treatment group samples, the dummy variable, Treat, equals 1, and for control group, the dummy variable, Treat, equals 0 [48]. Additionally, we used a time dummy variable (Post) to proxy whether the observation happens before or after the event. For observations made after 7 December 2018, Post equals 1, and 0 otherwise. The final independent variable (Treat*Post) is measured by the interaction of the treatment group dummy variable (Treat) and time dummy variable (Post).

#### 3.2.3. Control Variables

We also controlled other variables to avoid the possible influence on firm value as suggested by previous studies [49,50]. The description and measurement of all the variables are shown in Table 2.

#### 3.2.4. Regression Model

To test the mechanism how the high R&D investment intensity influence the AR caused by “4 + 7” procurement policy. We establish model (4) adopting DiD methodology.

(4) $$AR_{i,t}=\beta_{1}Treat+\beta_{2}Post+\beta_{3}Treat*Post+\sum \beta Controls_{i,t-1}+\varepsilon_{i,t-1}$$

In Equation (4), the coefficient $\beta_{3}$, is the net effect of high R&D investment on the AR caused by the “4 + 7” procurement policy.

DiD methodology requires that covariates be exogeneous to policy’s influence so that the difference is purely explained by the policy’s influence on the independent variable but not any covariates [51]. We used Propensity Score Matching (PSM) methodology to construct treated group and control group with comparable covariates so that except for the independent variable, the two groups were similar to each other. The matching process consists of the following steps: First, based on the selected covariates, we estimate the probability that a company is a high R&D investment company by running a logit regression. In this estimation function, we select the covariates which maximize function R^2^ and influence both the treated variable (TREAT) and the dependent variable (AR). By considering these covariates in the matching process, we meet the parallel trend assumption, that DiD analysis outcome should not be related to the covariates. We select firm size, firm age, leverage ratios, and profitability ratios as the covariates in the estimation function and the logit regression outcome used in the next step is shown in Table 3.

Second, based on the probability that every sample becomes a high R&D investment one, we employed a kernel function to weigh and match the samples. Last, following the method used by Fang, we tested the parallel trend to verify the effectiveness of the matching by comparing the treated group and the control group. The results are shown in Panel B of Table 3.

## 4. Results

### 4.1. Descriptive Statistics

Table 4 lists the mean, standard deviation, maximum and minimum values of all variables. As shown in the table, the mean values of AR of all samples, the treated group and the control group are −0.003, −0.004, −0.002, respectively, and the standard deviations of AR of the full sample, the treated group and the control group are 0.02, 0.023, 0.019, respectively. The coefficient of variation is bigger than 5, showing that for different pharmaceutical companies, the ARs raised by the “4 + 7” procurement policy are varied. From a general point of view, the trend of pharmaceutical stocks is generally lower than the market index, showing that the “4 + 7” procurement policy did negatively impact the pharmaceutical industry. The difference between treated group and control group also shows that high R&D investment companies face more AR decrease. The mean value of R&D intensity is 5.624, and the standard deviation is 5.293, showing that differences exist among samples in R&D investment. The mean value of R&D intensity (RDI) is 49.87 for the treated group and 3.647 for the control group. The difference in RDI also verifies our matching process.

Table 5 lists the correlations of all variables. The correlation between RDI and AR is significantly negative (−0.041), which shows the negative influence of high R&D investment on firm value under the impact of “4 + 7” procurement policy. Table 6 shows the multicollinearity test outcome of the variables in this study. The biggest variance inflation factor (VIF) is 2.35 and the average value is 1.51. All the VIFs are far below the rule-of-thumb cutoff of 10. Therefore, there is no sign of multicollinearity, and the variables are ready for further analysis.

### 4.2. Main Effect

Table 7 presents the stepwise regression results using the DiD methodology. The dimension of the dependent variable in the final model is the percentage (%). From Model 1 to Model 3, we add control variables, Treat, and Treat*Post, in turn. From Model 4 to Model 6, we add the time fixed effect to Model 1 to Model 3 in turn. Our study is to investigate the influence of high R&D investment on negative market reaction during the announcement of the “4 + 7” procurement policy. As Table 7 shows, the results of Model 2 and Model 5 preliminarily show that high R&D investment intensity has a significant negative impact on the AR caused by the “4 + 7” procurement policy at the confidence levels of 5% (β = −0.1579, *p* < 0.05 in Model 2) and 10% (β = −0.1217, *p* < 0.1 in Model 5), respectively. Model 3 and Model 6 show the impact of high R&D investment intensity on AR caused by the “4 + 7” procurement policy, which is also the regression result of DiD regression in the equation above. According to the DiD methodology [39,41], the coefficient of the interactive term Treat*Post reflects the difference between high R&D investment intensity or low R&D investment intensity and AR before and after the implementation of the “4 + 7” procurement policy. Table 7 shows that no matter whether controlling the time fixed effect or not, the interaction term is significantly negative to the AR at the 5% confidence level in both Model 3 and Model 6 (β = −0.3596, *p* < 0.05 in Model 3; β = −0.2978, *p* < 0.05 in Model 6). All regression results were consistent with the results of descriptive statistics. The two coefficients of the interaction term are both around −0.3, indicating that after the implementation of the “4 + 7” procurement policy, the AR of firms with high R&D investment intensity are 0.3% lower than that of firms with low R&D investment intensity. Hypothesis b is supported.

### 4.3. Robustness Check

In order to ensure the accuracy and reliability of the study, we also performed the following robustness checks.

**Alternative dependent variables and methods.** To be specific, we calculate the CARs for 1 day, 5 days, and 10 days around the event day, respectively. The new dependent variables are named CAR [−1,1], CAR [−1,10], CAR [−5,5], and CAR [−10,10]. Using the traditional event analysis method, the regression results with new dependent variables are shown in Table 8. The coefficient of the Treat is negative and significant (β = −0.0616, *p* < 0.05 in Model 1; β = −0.0416, *p* < 0.1 in Model 2; β = −0.0271, *p* < 0,05 in Model 3; β = −0.0649, *p* < 0.01 in Model 4), indicating that the research results are not affected by the window period used in CAR calculating process.

**Alternative samples.** Specifically, we also use the full sample without PSM to verify the above model. The regression results are shown in Table 9. The key variables in all models present the same effect on the dependent variable with the variables after PSM. The coefficient of Treat is negative and significant (β = −0.1508, *p* < 0.05 in Model 2; β = −0.1166, *p* < 0.1 in Model 5), and that of the interactive term Treat*Post is also negative and significant (β = −0.3721, *p* < 0.05 in Model 3; β = −0.3117, *p* < 0.05 in Model 6).

**Alternative independent variables.** We use the proportion of R&D personnel to measure whether the firm has high R&D intensity. To be specific, we use a new treatment variable, Treat2. For firms with R&D personnel accounting for more than 75% quantile, Treat2 equals 1. The regression results of the above models are shown in Table 10. The regression coefficients of key variables in all models are consistent with the previous results. The coefficient of Treat2 is negative and significant (β = −0.1602, *p* < 0.05 in Model 2; β = −0.1338, *p* < 0.05 in Model 5), and that of the interactive term Treat2*Post is also negative and significant (β = −0.2499, *p* < 0.1 in Model 3; β = −0.2082, *p* < 0.1 in Model 6).

### 4.4. Further Study

We find that under the background of “4 + 7” procurement policy, compared with firms with low R&D investment intensity, firms with high R&D investment intensity suffer more loss in firm value. In this part, we further examine the situational factors that cause the AR changes of pharmaceutical firms with high R&D intensity after the “4 + 7” procurement policy.

**The moderating role of firm size.** Studies have shown that there is a relationship between R&D investment and firm performance with the firm size [52]. The growth of firm size is often accompanied by the accumulation of capital, and capital accumulation leads to higher R&D investment intensity. Therefore, big firms bear greater R&D investment risks than small firms. In addition, when the R&D investment intensity is the same, the labor, sales, and management costs faced by big firms will be much higher than those faced by small firms. In the face of great changes in the industry, it is difficult for big firms to respond quickly in a short time, and the original advantages have become unfavorable factors in competition. Secondly, firm size significantly affects organizational inertia. With firm size growing bigger, organizational inertia grows and limits the corporate perception of changes in the external environment. Facing industry changes, big firms may respond more slowly. Thirdly, with the firm size growing larger, investors’ expectations for R&D investment grow higher. When the environment changes, the high R&D may become a “liability” for big firms. Finally, the capital market theory holds that small firms are less exposed to media and analysts than big firms, which leads to the market tending to generate more response to events of big firms. Therefore, we chose firm size as a moderating variable. Table 11 shows the regression results for testing the moderating effect. Model 1 and 2 respectively show the DiD regression results of full sample with and without time fixed effect, and model 3 and 4 respectively show the DiD regression results of the sample after PSM with and without time fixed effect. In order to prevent the possible interaction between firm size and R&D investment before and after policy implementation, we added Post*SIZE and Treat*SIZE as control variables when testing the moderating effect. The results show that the regression coefficient of Treat*Post*SIZE is significantly negative (β = −0.2094, *p* < 0.05 in Model 1; β = −0.2486, *p* < 0.01 in Model 2; β = −0.2250, *p* < 0.05 in Model 3; β = −0.2658, *p* < 0.01 in Model 4) regardless of the time fixed effect, which means that the bigger the firm size, the greater the negative impact of high R&D investment intensity on the AR after the “4 + 7” procurement policy.

**Figure 1:**
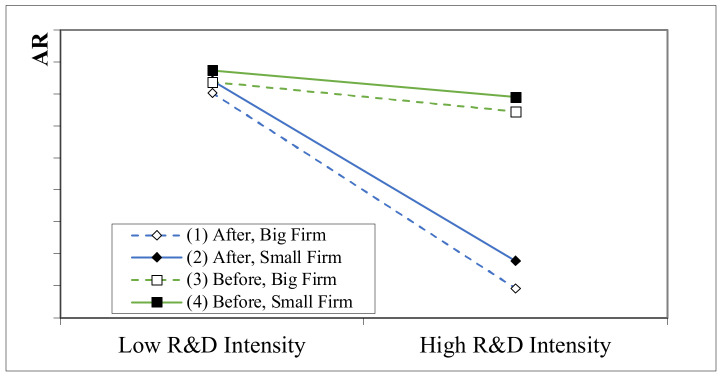
The moderating effect of firm size.

In order to present the moderating role of firm size more vividly, we illustrate the negative impact of high R&D investment intensity on the AR after the “4 + 7” procurement policy for different levels of the firm size. Following the method recommended by Aiken et al. [53], we created the image shown in Figure 1. It can be found that the slopes of big firms are steeper, compared with those of small firms, which indicates that high R&D investment intensity has a greater negative impact on big firms after the “4 + 7” procurement policy. The figure also shows that after the implementation of the “4 + 7” procurement policy, the high R&D investment intensity has a greater negative impact on AR caused by the “4 + 7” procurement policy.

**The moderating effect of pharmaceutical firm type.** Many people believe that the “4 + 7” procurement policy not only adjusts the market structure of the generic pharmaceutical industry but also forces firms to invest more in R&D and the production of innovative drugs. A consensus for China’s innovative pharmaceutical firms is that their R&D capacity of the original drug is weak. “Me too” innovative drug firms refer to the firms which make improvements to original drugs instead of developing original on their own. These innovative drug firms depend their R&D activity heavily on original drugs. What is the impact of the “4 + 7” procurement policy on “me too” innovative drug firms? We take pharmaceutical firm type as a moderating variable in our model and the test results are shown in Table 12. Models 1 and 2 are the DiD regression results of the whole sample with and without time fixed effect, respectively. Models 3 and 4 are the DiD regression results of the sample after PSM with and without time fixed effect, respectively. In order to prevent the possible interaction between firm type and R&D investment intensity before and after policy implementation, we add POST*ID and Treat*ID as control variables when testing the moderating effect. The results show that the coefficient of Treat*Post*ID is negative and significant (β = −0.7885, *p* < 0.05 in Model 1; β = −0.7136, *p* < 0.01 in Model 2; β = −0.9164, *p* < 0.01 in Model 3; β = −0.8594, *p* < 0.01 in Model 4), indicating that for innovative pharmaceutical firms in China, high R&D intensity has a greater negative impact on AR caused by the “4 + 7” procurement policy. The phenomenon can be explained by that most Chinese innovative drug firms are not capable of developing original drugs even though they have invested heavily in innovation. Most so-called new drugs are still “me too” drugs developed from original drugs. “Me too” drugs are expected to face more competitors than original drugs. The profit from “me too” drugs cannot last long, because “me too” drugs are not protected by intellectual property right like original drugs. In the context of the “4 + 7” procurement policy, Chinese innovative drug firms will be more affected by the “liability” characteristic of high R&D investment.

## 5. Conclusions

This study investigates whether the major policy of the pharmaceutical industry, the “4 + 7” volume-based procurement policy, promotes the R&D investment of pharmaceutical firms. By employing event analysis and the DiD method, we empirically test the influence of high R&D investment on firm value before and after the policy implementation. The results show that firms with high R&D investment intensity bear significantly lower ARs around the event day than those with low R&D investment intensity. The DiD analysis based on the AR before and after the event shows that after the announcement of the “4 + 7” procurement bidding results, compared with that of pharmaceutical firms with low R&D intensity, the AR of pharmaceutical firms with high R&D intensity were reduced by about 0.3% during event window period. The results support the hypothesis that high R&D investment intensity has a negative impact on firm value, that is, the “4 + 7” procurement policy inhibits the R&D investment of pharmaceutical firms. Furthermore, we study the moderating effect of firm size and pharmaceutical firm type on the relationship between R&D investment and firm value. In the context of the “4 + 7” procurement policy, the greater the firm size, the greater the negative impact of high R&D investment on the AR. The heavy R&D investment has a greater negative impact on AR for innovative pharmaceutical firms than for other types of pharmaceutical firms. The conclusions are contrary to the original intention of the “4 + 7” procurement policy to promote the innovation and transformation of pharmaceutical firms.

We put forward three solutions to mitigate the possible negative influence of the “4 + 7” procurement policy based on our study. First, it is necessary to increase policy incentives for R&D investment in the pharmaceutical industry to alleviate the inhibitory effect on R&D investment caused by the implementation of the “4 + 7” procurement policy. Secondly, it is necessary to support innovative pharmaceutical firms and big firms in R&D activities to form the innovative backbone of the pharmaceutical industry. Although large pharmaceutical firms and innovative pharmaceutical firms have relatively strong risk response capability, they are not yet capable of inventing original drugs. The “me-too” drugs developed are weak in competitions in mass procurement biddings. The problems brought by high R&D investment still act as “liabilities” for large pharmaceutical firms and innovative firms. Finally, it is necessary to change the current situation that pharmaceutical companies focus their R&D forces on me-too drugs. By reforming the pharmaceutical industry system, accelerating drug approval, and strengthening intellectual property rights protection, the government should encourage firms to engage more in the R&D of original drugs. The growth of innovative pharmaceutical firms focusing on original drugs finally will cooperate with the volume-based procurement policy to provide the better quality and cheaper prices, to promote industry transformation and upgrading, and to ease the burden of the health care system.

## Figures and Tables

**Table 1:** *t*-test of CAR.

Event Window	CAR	t-Value
(−10, 0)	−0.0396 ***	−9.680
(−10, 1)	−0.0478 ***	−10.500
(−10, 2)	−0.0468 ***	−9.750
(−10, 3)	−0.0516 ***	−10.550
(−10, 4)	−0.0566 ***	−11.720
(−10, 5)	−0.0673 ***	−13.010
(−10, 6)	−0.0726 ***	−14.000
(−10, 7)	−0.0672 ***	−13.200
(−10, 8)	−0.0739 ***	−13.980
(−10, 9)	−0.0617 ***	−11.800
(−10, 10)	−0.0578 ***	−10.720
(−5, 0)	−0.0123 **	−3.540
(−5, 1)	−0.0205 ***	−5.490
(−5, 2)	−0.0196 ***	−4.900
(−5, 3)	−0.0246 ***	−5.910
(−5, 4)	−0.0300 ***	−7.270
(−5, 5)	−0.0405 ***	−9.070
(−2, 0)	−0.0246 ***	−8.350
(−2, 1)	−0.032 ***	−9.920
(−2, 2)	−0.0320 ***	−8.900
(−2, 3)	−0.0371 ***	−9.860
(−2, 4)	−0.0427 ***	−11.430
(−2, 5)	−0.0531 ***	−12.480
(−2, 6)	−0.0586 ***	−13.400
(−2, 7)	−0.0532 ***	−12.440
(−2, 8)	−0.0598 ***	−12.890
(−2, 9)	−0.0476 ***	−10.070
(−2, 10)	−0.0435 ***	−8.560
(−1, 0)	−0.0317 ***	−11.460
(−1, 1)	−0.0400 ***	−12.580
(−1, 2)	−0.0392 ***	−11.420
(−1, 3)	−0.0443 ***	−12.070
(−1, 4)	−0.0499 ***	−13.910
(−1, 5)	−0.0604 ***	−14.650
(−1, 6)	−0.0659 ***	−15.430
(−1, 7)	−0.0606 ***	−14.310
(−1, 8)	−0.0672 ***	−14.410
(−1, 9)	−0.0550 ***	−11.560
(−1, 10)	−0.0509 ***	−9.950

Notes: significance levels: ** *p* < 0.01; *** *p* < 0.001.

**Table 2:** Variable description and measurement.

Variable Type	Variables	Variable Name	Description
Dependent Variable	Firm value	AR	The CAR during the event window (−10,10)
Independent Variables	Treatment variable	Treat	Dummy variable, coded as 1 if R&D intensity of the firm is above 75% percentile, and 0 otherwise
	Time variable	Post	Dummy variable, coded as 1 if a firm is observed after 7 December 2018
	R&D intensity	RDI	R&D input/Sales revenue (2017)
	Proportion of R&D personnel	RDPI	Number of R&D personnel/total number of employees (2017)
Control Variables	Firm age	AGE	Time since establishment of the company (2017)
	Tobin’s Q	Tobinsq	Market value/Asset value (2017)
	ROA	ROA	Net profit/Total asset (2017)
	Debt to asset ratio	Lev	Debt/Asset (2017)
	Liquidity ratio	Cash Ratio	Cash and cash equivalents/current liabilities (2017)
	Return on fixed asset	ROF	Net profit/fixed asset (2017)
	Return on investment	ROI	Investment return/(Long-term equity investment + held-to-maturity investment + transactional financial assets + available-for-sale financial assets + derivative financial assets) (2017)
	Bid winner	Target	1, if the firm won the bid on 7 December 2018; 0 otherwise
	Certificated high-tech	Tech	1, if the firm is certificated high-tech firm according to the government; 0 otherwise (2017)
	Generic drug concept stock	AND	1, if the firm is a generic drug concept stock; 0, otherwise (2017)
	Innovative drug concept stock	ID	1, if the firm is an innovative drug concept stock; 0 otherwise (2017)
	Firm ownership	Ownership	1, if the firm is state-owned; 0, otherwise (2017)
	Firm size	SIZE	Natural logarithm of total asset (2017)

**Table 3:** Propensity score matching procedure.

**Panel A: Logit Model Used to Find Propensity Scores**				
**Variables**			**Independent Variable = Treat**	
AGE			−0.030 ** (0.015)	
Tobinsq			484.308 *** (43.716)	
ROA			1.607 (1.682)	
Lev			0.112 ** (0.035)	
Cash Ratio			−0.980 *** (0.194)	
ROF			0.104 *** (0.036)	
ROI			−0.432 *** (0.195)	
Ownership			−0.577 *** (0.186)	
SIZE			0.777 *** (0.194)	
Constant			−9.331 *** (1.840)	
N			1550	
Pseudo R^2^			0.15	
**Panel B: Test of the effectiveness of the propensity score matches**				
Variable	Average, Treated group	Average, Control group	% bias	*t*-test
AGE	18.5	−0.000	−3.4	−0.66
Tobinsq	0.003	18.421	−3.3	−0.60
ROA	0.088	0.003	−3.4	0.48
Lev	0.275	0.082	9.0	1.71 *
Cash Ratio	1.527	3.455	9.6	1.84 *
ROF	0.564	1.579	−2.3	−0.47
ROI	0.745	0.101	3.1	0.57
Ownership	0.142	0.728	−1.7	−0.35
SIZE	9.565	0.147	−6.0	−1.15

Notes: standard errors in parentheses; * *p* < 0.1, ** *p* < 0.05, *** *p* < 0.01.

**Table 4:** Descriptive statistics.

Variables	Full Sample					Treated Group					Control Group				
	N	Mean	SD	Min	Max	N	Mean	SD	Min	Max	N	Mean	SD	Min	Max
AR	3948	−0.003	0.02	−0.116	0.109	739	−0.004	0.023	−0.101	0.098	2423	−0.002	0.019	−0.116	0.106
Treat	3948	0.266	0.442	0	1	739	1	0	1	1	2423	0	0	0	0
RDI	3864	5.624	5.293	0.035	49.87	718	11.62	8.357	6.75	49.87	2423	3.647	1.618	0.035	6.64
AGE	3906	18.76	4.7	7.699	36.48	739	18.5	4.978	9.866	36.48	2423	19.28	4.471	7.953	29.85
Tobinsq	3948	0.003	0.002	0	0.017	739	0.003	0.003	0	0.012	2423	0.002	0.002	0	0.013
ROA	3906	0.078	0.058	−0.245	0.34	739	0.088	0.066	−0.095	0.282	2423	0.073	0.059	−0.245	0.34
Lev	3906	0.281	0.158	0.042	0.886	739	0.275	0.144	0.042	0.636	2423	0.3	0.166	0.045	0.886
Cash Ratio	3906	1.299	1.955	0.017	19.04	739	1.527	3.217	0.063	19.04	2423	1.073	1.253	0.017	7.058
ROF	3906	0.571	0.649	−0.81	4.583	739	0.564	0.535	−0.645	2.176	2423	0.497	0.538	−0.81	3.2
ROI	3255	0.576	1.794	−0.729	14.57	739	0.745	2.48	−0.252	14.57	2423	0.541	1.56	−0.729	10.58
Target	3948	0.032	0.176	0	1	739	0.114	0.318	0	1	2423	0.017	0.131	0	1
Tech	3906	0.253	0.435	0	1	739	0.256	0.437	0	1	2423	0.243	0.429	0	1
AND	3948	0.117	0.321	0	1	739	0.242	0.429	0	1	2423	0.078	0.268	0	1
ID	3948	0.277	0.447	0	1	739	0.441	0.497	0	1	2423	0.26	0.439	0	1
Ownership	3906	0.188	0.391	0	1	739	0.142	0.349	0	1	2423	0.243	0.429	0	1
SIZE	3906	9.519	0.435	8.787	10.79	739	9.565	0.47	8.801	10.79	2423	9.609	0.413	8.787	10.55

**Table 5:** Correlations.

Variables	1	2	3	4	5	6	7	
1. AR	1.000							
2. Treat	−0.041 *	1.000						
3. AGE	0.003	−0.072 ***	1.000					
4. Tobinsq	−0.008	0.299 ***	−0.085 ***	1.000				
5. ROA	−0.011	0.103 ***	0.089 ***	0.453 ***	1.000			
6. Lev	−0.021	−0.067 ***	0.194 ***	−0.417 ***	−0.390 ***	1.000		
7. Cash Ratio	0.009	0.100 ***	−0.079 ***	0.262 ***	0.317 ***	−0.429 ***	1.000	
8. ROF	0.006	0.052 **	0.053 **	0.481 ***	0.768 ***	−0.341 ***	0.341 ***	
9. ROI	−0.040 *	0.047 **	0.124 ***	0.033	0.393 ***	−0.046 **	0.127 ***	
10. Target	−0.022	0.208 ***	−0.090 ***	−0.014	0.049 **	0.035	−0.027	
11. Tech	−0.005	0.013	−0.124 ***	0.116 ***	−0.009	−0.122 ***	−0.086 ***	
12. AND	-0.033	0.217 ***	0.014	−0.061 ***	0.032	0.097 ***	−0.069 ***	
13. ID	0.009	0.167 ***	−0.110 ***	−0.063 ***	−0.037*	0.106 ***	−0.118 ***	
14. Ownership	−0.015	−0.103 ***	0.180 ***	−0.127 ***	−0.049 **	0.203 ***	0.001	
15. SIZE	−0.033	−0.044 *	0.163 ***	−0.327 ***	0.030	0.423 ***	−0.218 ***	
	**8**	**9**	**10**	**11**	**12**	**13**	**14**	**15**
8. ROF	1.000							
9. ROI	0.248 ***	1.000						
10. Target	0.051 **	−0.009	1.000					
11. Tech	−0.119 ***	0.027	−0.116 ***	1.000				
12. AND	0.026	0.029	0.455 ***	−0.207 ***	1.000			
13. ID	−0.066 ***	−0.019	0.088 ***	0.027	0.236 ***	1.000		
14. Ownership	0.024	0.142 ***	−0.108 ***	−0.191 ***	−0.092 ***	−0.174 ***	1.000	
15. SIZE	0.106 ***	0.059 ***	0.115 ***	−0.332 ***	0.274 ***	0.241 ***	0.227 ***	1.000

Notes: Significance levels: * *p* < 0.05; ** *p* < 0.01; *** *p* < 0.001.

**Table 6:** Variance inflation factor test.

Variables	VIF	1/VIF
ROA	2.35	0.425
ROF	1.96	0.510
Lev	1.80	0.556
SIZE	1.77	0.564
Tobinsq	1.65	0.606
AND	1.46	0.686
Cash Ratio	1.40	0.712
Target	1.31	0.763
Treat	1.30	0.768
ROI	1.28	0.779
ID	1.24	0.803
Ownership	1.23	0.812
Tech	1.21	0.829
AGE	1.14	0.878
Mean VIF	1.51	

**Table 7:** PSM-DID regression results.

Variables	(1)	(2)	(3)	(4)	(5)	(6)
	AR	AR	AR	AR	AR	AR
AGE	0.0045	0.0064	0.0046	0.0022	0.0019	0.0020
	(0.007)	(0.007)	(0.007)	(0.006)	(0.006)	(0.006)
Tobinsq	−44.7663 **	−29.1016	−39.7596 *	−39.2848 **	−29.5914	−30.3089
	(21.427)	(18.280)	(21.694)	(18.664)	(19.289)	(19.329)
ROA	0.0976	−0.5764	0.0714	−0.0707	−0.0498	−0.0212
	(0.777)	(0.746)	(0.783)	(0.683)	(0.687)	(0.683)
Lev	−0.1204	−0.1511	−0.1066	−0.1326	−0.1255	−0.1289
	(0.211)	(0.177)	(0.213)	(0.181)	(0.181)	(0.180)
Cash Ratio	−0.0121	0.0009	−0.0090	−0.0079	−0.0043	−0.0046
	(0.023)	(0.012)	(0.023)	(0.019)	(0.019)	(0.019)
ROF	0.2279 **	0.1733 **	0.2255 **	0.2036 **	0.1888 **	0.1875 **
	(0.094)	(0.083)	(0.096)	(0.086)	(0.086)	(0.086)
ROI	−0.0630 ***	−0.0432 ***	−0.0618 ***	−0.0609 ***	−0.0595 ***	−0.0593 ***
	(0.021)	(0.011)	(0.021)	(0.018)	(0.018)	(0.018)
Target	−0.1440	−0.0611	−0.1160	−0.1465	−0.1048	−0.1091
	(0.199)	(0.099)	(0.197)	(0.156)	(0.157)	(0.156)
Tech	−0.0273	−0.0857	−0.0264	−0.0038	−0.0031	−0.0058
	(0.073)	(0.064)	(0.073)	(0.059)	(0.059)	(0.059)
AND	−0.0393	−0.1448 *	−0.0300	−0.0116	−0.0043	−0.0055
	(0.107)	(0.086)	(0.105)	(0.083)	(0.082)	(0.082)
ID	0.1487 **	0.1484 ***	0.1517 **	0.1576 ***	0.1641 ***	0.1633 ***
	(0.066)	(0.055)	(0.067)	(0.054)	(0.054)	(0.053)
Ownership	0.0069	−0.0388	0.0027	0.0150	0.0171	0.0149
	(0.071)	(0.058)	(0.071)	(0.059)	(0.059)	(0.059)
SIZE	−0.2304 **	−0.2273 ***	−0.2342 ***	−0.2066 ***	−0.2025 ***	−0.2005 ***
	(0.090)	(0.077)	(0.091)	(0.076)	(0.076)	(0.076)
Treat		−0.1579 **	0.0628		*−*0.1217 *	0.0265
		(0.070)	(0.115)		(0.067)	(0.096)
Post			−0.0918			2.4567 ***
			(0.059)			(0.158)
Treat* Post			−0.3596 **			−0.2978 **
			(0.154)			(0.127)
_cons	1.9290 **	1.9152 ***	2.0133 **	0.0611	0.0231	−0.0175
	(0.861)	(0.733)	(0.868)	(0.740)	(0.738)	(0.736)
time dummies	No	No	No	Yes	Yes	Yes
*N*	3162	3162	3162	3162	3162	3162
chi2	26.7636	93.6968	40.6066	1.3 × 10^3^	1.3 × 10^3^	1.3 × 10^3^

Notes: Standard errors in parentheses; * *p* < 0.1, ** *p* < 0.05, *** *p* < 0.01.

**Table 8:** Robustness checks on event study.

Variables	(1)	(2)	(3)	(4)
	CAR [−10,10]	CAR [−5,5]	CAR [−1,1]	CAR [−1,10]
AGE	0.0014	0.0006	0.0024 ***	0.0019 *
	(0.002)	(0.001)	(0.001)	(0.001)
ROA	−0.1320	−0.2717 **	−0.1619 *	−0.4019 **
	(0.157)	(0.134)	(0.090)	(0.173)
Lev	−0.0307	−0.0376	−0.0337	−0.0461
	(0.042)	(0.035)	(0.027)	(0.043)
Cash Ratio	−0.0005	−0.0006	0.0008	0.0002
	(0.003)	(0.002)	(0.002)	(0.003)
ROF	0.0149	0.0202 *	0.0085	0.0127 *
	(0.010)	(0.011)	(0.007)	(0.008)
ROI	−0.0087 ***	−0.0044	−0.0032	−0.0027
	(0.003)	(0.003)	(0.003)	(0.003)
Target	−0.0276	−0.0234 *	−0.0094	−0.0332
	(0.027)	(0.013)	(0.017)	(0.026)
Tech	−0.0244	−0.0170	−0.0167	−0.0282
	(0.019)	(0.015)	(0.014)	(0.021)
AND	0.0210	0.0118	−0.0130 *	−0.0058
	(0.013)	(0.010)	(0.007)	(0.012)
ID	−0.0041	0.0062	−0.0024	−0.0019
	(0.013)	(0.012)	(0.009)	(0.012)
Ownership	−0.0285 *	−0.0205	−0.0164 *	−0.0392 ***
	(0.015)	(0.013)	(0.009)	(0.013)
Treat	−0.0616 **	−0.0416 *	−0.0271 **	−0.0649 ***
	(0.024)	(0.023)	(0.013)	(0.021)
_cons	0.2070	0.1698	0.0984	0.3348 ***
	(0.138)	(0.118)	(0.085)	(0.119)
*N*	155	155	155	155
*R* ^2^	0.158	0.163	0.239	0.298
adj. *R*^2^	0.087	0.092	0.175	0.239

Notes: Standard errors in parentheses; * *p* < 0.1, ** *p* < 0.05, *** *p* < 0.01.

**Table 9:** Robustness checks on full sample.

Variables	(1)	(2)	(3)	(4)	(5)	(6)
	AR	AR	AR	AR	AR	AR
AGE	0.0042	0.0056	0.0040	0.0013	0.0009	0.0010
	(0.007)	(0.007)	(0.007)	(0.006)	(0.006)	(0.006)
Tobinsq	−45.6390 ***	−24.5801 *	−40.0337 **	−45.4149 ***	−37.0687 ***	−37.5037 ***
	(15.122)	(14.943)	(15.972)	(11.892)	(12.807)	(12.945)
ROA	0.7377	−0.1865	0.7423	0.6058	0.6285	0.6554
	(0.671)	(0.692)	(0.674)	(0.581)	(0.584)	(0.579)
Lev	−0.1874	−0.1885	−0.1723	−0.1939	−0.1867	−0.1880
	(0.209)	(0.181)	(0.211)	(0.180)	(0.180)	(0.179)
Cash Ratio	−0.0236	−0.0033	−0.0203	−0.0234	−0.0202	−0.0198
	(0.020)	(0.012)	(0.020)	(0.016)	(0.016)	(0.016)
ROF	0.1077 *	0.0775	0.0986 *	0.0851	0.0725	0.0705
	(0.056)	(0.051)	(0.056)	(0.052)	(0.052)	(0.052)
ROI	−0.0602 ***	−0.0407 ***	−0.0589 ***	−0.0581 ***	−0.0567 ***	−0.0565 ***
	(0.021)	(0.012)	(0.021)	(0.018)	(0.018)	(0.018)
Target	−0.1229	−0.0712	−0.0949	−0.1392	−0.0989	−0.1031
	(0.200)	(0.105)	(0.197)	(0.157)	(0.158)	(0.156)
Tech	−0.0450	−0.1027	−0.0467	−0.0264	−0.0259	−0.0291
	(0.071)	(0.063)	(0.071)	(0.057)	(0.057)	(0.057)
AND	−0.0442	−0.1323	−0.0374	−0.0110	−0.0031	−0.0063
	(0.105)	(0.081)	(0.104)	(0.082)	(0.082)	(0.082)
ID	0.1384 **	0.1335 **	0.1414 **	0.1460 ***	0.1519 ***	0.1514 ***
	(0.066)	(0.056)	(0.066)	(0.053)	(0.053)	(0.053)
Ownership	0.0108	−0.0343	0.0082	0.0226	0.0254	0.0228
	(0.071)	(0.060)	(0.071)	(0.059)	(0.059)	(0.059)
SIZE	−0.2032 **	−0.1948 ***	−0.2071 **	−0.1954 ***	−0.1933 ***	−0.1915 ***
	(0.083)	(0.074)	(0.084)	(0.072)	(0.072)	(0.072)
Treat		−0.1508 **	0.0752		−0.1166 *	0.0400
		(0.071)	(0.114)		(0.067)	(0.095)
Post			−0.0929			2.4606 ***
			(0.059)			(0.156)
Treat* Post			−0.3721 **			−0.3117 **
			(0.149)			(0.123)
_cons	1.7160 **	1.6438 **	1.8065 **	0.0192	0.0041	−0.0373
	(0.787)	(0.694)	(0.795)	(0.688)	(0.687)	(0.685)
time dummies	No	No	No	Yes	Yes	Yes
*N*	3255	3255	3255	3255	3255	3255
chi2	26.8702	65.4635	42.5369	1.3 × 10^3^	1.3 × 10^3^	1.3 × 10^3^

Notes: Standard errors in parentheses * *p* < 0.1, ** *p* < 0.05, *** *p* < 0.01.

**Table 10:** Robustness checks on alternative independent variables.

Variables	(1)	(2)	(3)	(4)	(5)	(6)
	AR	AR	AR	AR	AR	AR
AGE	0.0042	0.0032	0.0033	0.0013	0.0009	0.0009
	(0.007)	(0.007)	(0.007)	(0.006)	(0.006)	(0.006)
Tobinsq	−45.6390 ***	−44.2223 ***	−45.9828 ***	−45.4149 ***	−43.7701 ***	−44.5657 ***
	(15.122)	(15.146)	(15.051)	(11.892)	(12.007)	(11.981)
ROA	0.7377	0.6790	0.6733	0.6058	0.5734	0.5784
	(0.671)	(0.672)	(0.677)	(0.581)	(0.583)	(0.581)
Lev	−0.1874	−0.1766	−0.1704	−0.1939	−0.1760	−0.1793
	(0.209)	(0.209)	(0.212)	(0.180)	(0.180)	(0.179)
Cash Ratio	−0.0236	−0.0152	−0.0145	−0.0234	−0.0164	−0.0158
	(0.020)	(0.020)	(0.020)	(0.016)	(0.016)	(0.016)
ROF	0.1077 *	0.1097 **	0.1121 **	0.0851	0.0894 *	0.0906 *
	(0.056)	(0.056)	(0.056)	(0.052)	(0.052)	(0.052)
ROI	−0.0602 ***	−0.0641 ***	−0.0642 ***	−0.0581 ***	−0.0616 ***	−0.0617 ***
	(0.021)	(0.021)	(0.021)	(0.018)	(0.018)	(0.018)
Target	−0.1229	−0.1071	−0.1124	−0.1392	−0.1242	−0.1248
	(0.200)	(0.199)	(0.197)	(0.157)	(0.156)	(0.156)
Tech	−0.0450	−0.0419	−0.0422	−0.0264	−0.0260	−0.0264
	(0.071)	(0.071)	(0.071)	(0.057)	(0.057)	(0.057)
AND	−0.0442	−0.0540	−0.0518	−0.0110	−0.0207	−0.0215
	(0.105)	(0.105)	(0.105)	(0.082)	(0.082)	(0.082)
ID	0.1384 **	0.1491 **	0.1463 **	0.1460 ***	0.1537 ***	0.1527 ***
	(0.066)	(0.066)	(0.067)	(0.053)	(0.053)	(0.053)
Ownership	0.0108	0.0154	0.0149	0.0226	0.0231	0.0225
	(0.071)	(0.071)	(0.071)	(0.059)	(0.059)	(0.059)
SIZE	−0.2032 **	−0.2229 ***	−0.2277 ***	−0.1954 ***	−0.2110 ***	−0.2103 ***
	(0.083)	(0.083)	(0.085)	(0.072)	(0.072)	(0.072)
Treat2		−0.1602 **	−0.0314		−0.1338 **	−0.0274
		(0.073)	(0.103)		(0.058)	(0.083)
Post			−0.1045 *			2.4640 ***
			(0.060)			(0.156)
Treat2* Post			−0.2499 *			−0.2082 *
			(0.139)			(0.112)
_cons	1.7160 **	1.9406 **	2.0411 **	0.0192	0.1853	0.1581
	(0.787)	(0.791)	(0.803)	(0.688)	(0.692)	(0.690)
Time dummies	No	No	No	Yes	Yes	Yes
*N*	3255	3255	3255	3255	3255	3255
chi2	26.8702	32.0048	42.9573	1.3 × 10^3^	1.3 × 10^3^	1.3 × 10^3^

Notes: Standard errors in parentheses; * *p* < 0.1, ** *p* < 0.05, *** *p* < 0.01.

**Table 11:** Regression results of the moderating effect of firm size.

Variables	(1)	(2)	(3)	(4)
	AR	AR	AR	AR
AGE	0.0044	0.0018	0.0047	0.0026
	(0.007)	(0.006)	(0.007)	(0.006)
Tobinsq	−42.0477 ***	−40.0027 ***	−41.9173 *	−34.1553 *
	(16.166)	(13.148)	(21.832)	(19.460)
ROA	0.6925	0.5669	0.0309	−0.0930
	(0.672)	(0.576)	(0.781)	(0.679)
Lev	−0.1836	−0.2164	−0.1166	−0.1588
	(0.212)	(0.179)	(0.214)	(0.180)
Cash Ratio	−0.0247	−0.0252	−0.0145	−0.0113
	(0.020)	(0.016)	(0.023)	(0.019)
ROF	0.1090 *	0.0862	0.2374 **	0.2050 **
	(0.057)	(0.052)	(0.096)	(0.087)
ROI	−0.0566 ***	−0.0535 ***	−0.0594 ***	−0.0562 ***
	(0.021)	(0.018)	(0.021)	(0.018)
Target	−0.0616	−0.0604	−0.0821	−0.0647
	(0.197)	(0.157)	(0.197)	(0.156)
Tech	−0.0520	−0.0325	−0.0359	−0.0128
	(0.071)	(0.057)	(0.073)	(0.059)
AND	−0.0199	0.0122	−0.0124	0.0140
	(0.104)	(0.081)	(0.105)	(0.082)
ID	0.1557 **	0.1651 ***	0.1653 **	0.1759 ***
	(0.066)	(0.053)	(0.067)	(0.053)
Ownership	0.0356	0.0303	0.0316	0.0235
	(0.099)	(0.083)	(0.099)	(0.083)
SIZE	−0.1807 **	−0.1544 **	−0.2088 **	−0.1642 **
	(0.085)	(0.073)	(0.091)	(0.077)
Treat	0.0768	0.0411	0.0654	0.0300
	(0.114)	(0.094)	(0.115)	(0.095)
Post	−0.0914	2.4633 ***	−0.0900	2.4612 ***
	(0.060)	(0.155)	(0.060)	(0.158)
Treat* Post	−0.3709 **	−0.2972 **	−0.3543 **	−0.2843 **
	(0.150)	(0.122)	(0.154)	(0.126)
Post* SIZE	−0.0066	0.0094	−0.0079	0.0091
	(0.049)	(0.041)	(0.049)	(0.041)
Treat*SIZE	−0.0524	−0.0522	−0.0529	−0.0544
	(0.082)	(0.063)	(0.081)	(0.063)
Treat* Post*SIZE	−0.2094 **	−0.2486 ***	−0.2250 **	−0.2658 ***
	(0.100)	(0.082)	(0.104)	(0.084)
_cons	1.5404 *	−0.4011	1.7631 **	−0.3668
	(0.808)	(0.696)	(0.876)	(0.744)
time dummies	No	Yes	No	Yes
*N*	3255	3255	3162	3162
chi2	47.0473	1.4 × 10^3^	45.4735	1.3 × 10^3^

Notes: Standard errors in parentheses; * *p* < 0.1, ** *p* < 0.05, *** *p* < 0.01.

**Table 12:** Regression results of the moderating effect of innovative medicine-oriented firms.

Variables	(1)	(2)	(3)	(4)
	AR	AR	AR	AR
AGE	0.0039	0.0010	0.0042	0.0018
	(0.007)	(0.006)	(0.007)	(0.006)
Tobinsq	−36.1370 **	−33.2386 **	−37.6759 *	−28.9945
	(16.327)	(13.480)	(21.620)	(19.234)
ROA	0.7462	0.6392	0.1176	0.0041
	(0.671)	(0.576)	(0.781)	(0.680)
Lev	−0.1515	−0.1680	−0.0969	−0.1210
	(0.211)	(0.179)	(0.213)	(0.180)
Cash Ratio	−0.0233	−0.0222	−0.0114	−0.0063
	(0.020)	(0.016)	(0.023)	(0.019)
ROF	0.1009 *	0.0740	0.2218 **	0.1864 **
	(0.056)	(0.052)	(0.095)	(0.086)
ROI	−0.0555 ***	−0.0535 ***	−0.0596 ***	−0.0576 ***
	(0.021)	(0.018)	(0.021)	(0.018)
Target	−0.0594	−0.0762	−0.0947	−0.0958
	(0.198)	(0.156)	(0.198)	(0.156)
Tech	−0.0473	−0.0285	−0.0283	−0.0061
	(0.071)	(0.057)	(0.073)	(0.059)
AND	−0.0275	0.0019	−0.0193	0.0031
	(0.104)	(0.082)	(0.105)	(0.082)
ID	0.1323	0.1348 *	0.1295	0.1345 *
	(0.099)	(0.080)	(0.099)	(0.080)
Ownership	0.0131	0.0247	0.0070	0.0170
	(0.071)	(0.059)	(0.071)	(0.059)
SIZE	−0.1969 **	−0.1809 **	−0.2274 **	−0.1946 **
	(0.084)	(0.072)	(0.090)	(0.076)
Treat	0.0066	−0.0423	−0.0370	−0.0893
	(0.134)	(0.112)	(0.134)	(0.113)
Post	−0.1171 *	2.4432 ***	−0.1164 *	2.4393 ***
	(0.069)	(0.158)	(0.070)	(0.160)
Treat* Post	−0.0963	−0.0381	−0.0657	0.0001
	(0.180)	(0.152)	(0.182)	(0.152)
Post* ID	0.0866	0.0849	0.0859	0.0839
	(0.130)	(0.107)	(0.131)	(0.107)
ID*treat	0.1767	0.1839	0.3156	0.3286
	(0.236)	(0.192)	(0.248)	(0.201)
Treat* Post*ID	−0.7885 **	−0.7136 ***	−0.9164 ***	−0.8594 ***
	(0.316)	(0.257)	(0.332)	(0.270)
_cons	1.6960 **	−0.1510	1.9536 **	−0.0711
	(0.800)	(0.688)	(0.867)	(0.734)
time dummies	No	Yes	No	Yes
*N*	3255	3255	3162	3162
chi2	50.4716	1.3 × 10^3^	49.3844	1.3 × 10^3^

Notes: Standard errors in parentheses; * *p* < 0.1, ** *p* < 0.05, *** *p* < 0.01.

## Data Availability

The data that support the findings of this study are available from CSMAR and Wind databases. Restrictions apply to the availability of these data, which were used under license for this study. Data are available with the permission of CSMAR and Wind.
